# Presence of antioxidative agent, Pyrrolo[1,2-a]pyrazine-1,4-dione, hexahydro- in newly isolated *Streptomyces mangrovisoli* sp. nov.

**DOI:** 10.3389/fmicb.2015.00854

**Published:** 2015-08-20

**Authors:** Hooi-Leng Ser, Uma D. Palanisamy, Wai-Fong Yin, Sri N. Abd Malek, Kok-Gan Chan, Bey-Hing Goh, Learn-Han Lee

**Affiliations:** ^1^Biomedical Research Laboratory, Jeffrey Cheah School of Medicine and Health Sciences, Monash University MalaysiaBandar Sunway, Malaysia; ^2^Division of Genetics and Molecular Biology, Institute of Biological Sciences, Faculty of Science, University of MalayaKuala Lumpur, Malaysia; ^3^Biochemistry Program, Institute of Biological Sciences, Faculty of Science, University of MalayaKuala Lumpur, Malaysia

**Keywords:** *Streptomyces mangrovisoli*, novel taxa, antioxidant, DPPH, mangrove

## Abstract

A novel *Streptomyces*, strain MUSC 149^T^ was isolated from mangrove soil. A polyphasic approach was used to study the taxonomy of MUSC 149^T^, which shows a range of phylogenetic and chemotaxonomic properties consistent with those of the members of the genus *Streptomyces*. The diamino acid of the cell wall peptidoglycan was LL-diaminopimelic acid. The predominant menaquinones were identified as MK9(H_8_) and MK9(H_6_). Phylogenetic analysis indicated that closely related strains include *Streptomyces rhizophilus* NBRC 108885^T^ (99.2% sequence similarity), *S. gramineus* NBRC 107863^T^ (98.7%) and *S. graminisoli* NBRC 108883^T^ (98.5%). The DNA–DNA relatedness values between MUSC 149^T^ and closely related type strains ranged from 12.4 ± 3.3% to 27.3 ± 1.9%. The DNA G + C content was determined to be 72.7 mol%. The extract of MUSC 149^T^ exhibited strong antioxidant activity and chemical analysis reported identification of an antioxidant agent, Pyrrolo[1,2-a]pyrazine-1,4-dione, hexahydro-. These data showed that metabolites of MUSC 149^T^ shall be useful as preventive agent against free-radical associated diseases. Based on the polyphasic study of MUSC 149^T^, the strain merits assignment to a novel species, for which the name *S. mangrovisoli* sp. nov. is proposed. The type strain is MUSC 149^T^ (=MCCC 1K00699^T^=DSM 100438^T^).

## Introduction

Oxidative stress has been implicated in physiological aging which may contribute to the development of chronic diseases. The disequilibrium of oxidation status has been associated with development of neurodegenerative diseases which includes Parkinson’s disease and Alzheimer’s disease (Floyd and Hensley, 2002; Farooqui and Farooqui, 2009). In fact, oxidative stress is recognized to play a critical role in carcinogenesis as well. It is plausible that the accumulation of free radicals results in various modifications or damages to biological macromolecules such as protein, lipid, and DNA (Reuter et al., 2010). These unwanted, harmful effects then expedite DNA mutation and increase cancer risks. Therefore, the discovery of the antioxidants from natural resources has always sparked great interest of researchers (Lee et al., 1998).

The mangrove is an exclusive woody plant area of intertidal coasts in tropical and subtropical coastal regions. This ecosystem is among the world’s most prolific environments and produces commercial forest products, protects coastlines and supports coastal fisheries. Mangrove ecosystems are habitats of various flora and fauna of marine, freshwater and terrestrial species (Jennerjahn and Ittekkot, 2002). Recently, there has been increasing interest in exploitation of mangrove microorganism resources as the constant changes in factors such as salinity and tidal gradient in the mangrove ecosystems are consideration to be driving forces for metabolic pathway adaptations that could direct to the production of valuable metabolites (Hong et al., 2009; Lee et al., 2014a). Lately, numerous studies have discovered novel actinobacteria from the different mangrove environments globally, such as the isolation of *Streptomyces avicenniae* (Xiao et al., 2009), *S. xiamenensis* (Xu et al., 2009), *S. sanyensis* (Sui et al., 2011), *S. qinglanensis* (Hu et al., 2012), *S. pluripotens* (Lee et al., 2014b), and *S. gilvigriseus* (Ser et al., 2015).

Waksman and Henrici (1943) had proposed the genus *Streptomyces*; the genus *Streptomyces* is comprised of ca. 600 species with validly published names (http://www.bacterio.cict.fr/) at the time of writing (May 2015). Many members of this genus have made vital contributions to mankind due to their capabilities to produce various natural products (Berdy, 2005). These *Streptomyces*-derived secondary metabolites have attracted much attention from the community as they possess diverse bioactivities such as antibacterial, antifungal, antitumor, and antioxidant (Kaneko et al., 1989; Kim et al., 2008; Olano et al., 2009a; Saurav and Kannabiran, 2012; Thenmozhi and Kannabiran, 2012; Wang et al., 2013; Kumar et al., 2014; Khieu et al., 2015). Notably, some of the bioactivities described were associated with production of cyclic compounds such as cyclomarins and pyrrolizidines (Renner et al., 1999; Karanja et al., 2010; Fu and MacMillan, 2015).

In this study, this particular strain of *Streptomyces* was isolated from a mangrove soil located from the Tanjung Lumpur mangrove forest located in east coast of Peninsular Malaysia. With the polyphasic approach, it is revealed that MUSC 149^T^ represents a novel species of the *Streptomyces* genus, for which the name *S*. *mangrovisoli* sp. nov. is proposed. In our very initial attempt to explore the potential biological activity possessed by MUSC149^T^, antioxidant activity was examined. The result indicated that MUSC149^T^ extract exhibited a significant antioxidant property. To the best of our knowledge, the antioxidant activity of MUSC149^T^ has hitherto not been reported. The chemical analysis was then conducted to identify the chemical constituents present in the extract of MUSC149^T^. The outcomes derived from this research have provided a strong foundation for further in depth biological studies to be performed particularly focusing on free-radical associated diseases.

## Materials and Methods

### Isolation and Maintenance of Isolate

Strain MUSC 149^T^ was isolated from a soil sample collected at site MUSC-TLS1 (3° 48′ 3.2′′ N 103° 20′ 11.0′′ E), located in the mangrove forest of Tanjung Lumpur in the state of Pahang, Peninsular Malaysia, in December 2012. Topsoil samples of the upper 20-cm layer (after removing the top 2–3 cm) were collected and sampled into sterile plastic bags using an aseptic metal trowel, and stored at –20°C. Air-dried soil samples were ground with a mortar and pestle. Selective pretreatment of soil samples was performed using wet heat in sterilized water (15 min at 50°C; Takahashi et al., 1996). Five grams of the pretreated air-dried soil was mixed with 45 ml sterilized water and mill ground, spread onto the isolation medium ISP 2 (Shirling and Gottlieb, 1966) supplemented with cycloheximide (25 μg ml^-1^) and nystatin (10 μg ml^-1^), and incubated at 28°C for 14 days. Pure cultures of strain MUSC 149^T^ were isolated and maintained on slants of ISP 2 agar at 28°C and as glycerol suspensions (20%, v/v) at –20°C for long term preservation.

### Genomic and Phylogenetic Analyses

**FIGURE 1:**
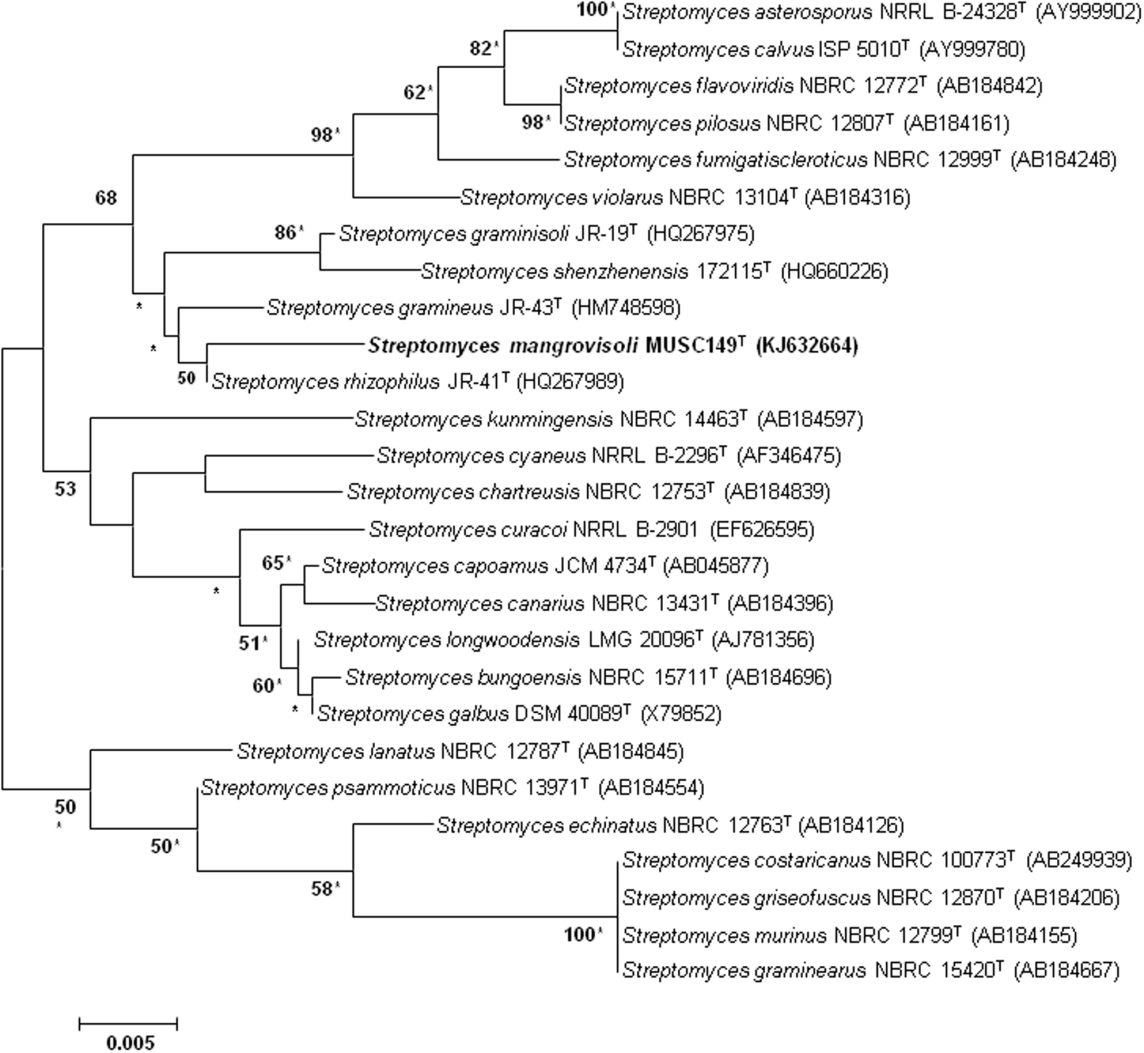
**Maximum-likelihood phylogenetic tree based on 1487 nucleotides of 16S rRNA gene sequence showing the relationship between strain MUSC 149^T^ and representatives of related taxa**. Numbers at nodes indicate percentages of 1000 bootstrap re-samplings, only values above 50% are shown. Bar, 0.005 substitutions per site. Asterisks indicate that the corresponding nodes were also recovered using the neighbor-joining tree-making algorithm.

The extraction of genomic DNA for PCR was performed as described by Hong et al. (2009). In short, approximately 0.5 g of each culture was suspended in TE buffer (0.5 ml) and ribolised for 30 s at a speed of 5.5 m/s following the addition of sterile glass beads (0.5 g, 100 mesh). The resultant preparations were extracted with an equal volume of chloroform: *iso*-amyl alcohol (24:1, v/v) and centrifuged at 15,000 g for 5 min at 4°C. The upper aqueous layers, which contained the DNA, were transferred to fresh tubes and used as template DNA. The amplification of 16S rRNA gene was performed according to Lee et al. (2014b). Briefly the PCR reactions were performed in a final volume of 50 μl according to protocol of SolGent^TM^ 2X Taq PLUS PCR Smart mix using the Kyratex PCR Supercycler (Kyratec, Australia) with the following cycling conditions: (i) 95°C for 5 min, (ii) 35 cycles of 94°C for 50 s, 55°C for 1 min and 72°C for 1 min 30 s; and (iii) 72°C for 8 min. The 16S rRNA gene sequence of strain MUSC 149^T^ was aligned with representative sequences of related type strains of the genus *Streptomyces* retrieved from the GenBank/EMBL/DDBJ databases using CLUSTAL-X software (Thompson et al., 1997). The alignment was verified manually and then used to generate phylogenetic tree. Phylogenetic trees were constructed with the maximum-likelihood (Felsenstein, 1981) (**Figure 1**) and neighbor-joining (Saitou and Nei, 1987) (Supplementary Figure S1) algorithms using MEGA version 5.2 (Tamura et al., 2011). Evolutionary distances for the neighbor-joining algorithm were computed using Kimura’s two-parameter model (Kimura, 1980). The EzTaxon-e server (http://eztaxon-e.ezbiocloud.net/; Kim et al., 2012) was used for calculations of sequence similarity. The stability of the resultant trees topologies were evaluated by using the bootstrap based on 1000 resampling method of Felsenstein (1985).

BOX-PCR fingerprint analysis was used to characterize strain MUSC 149^T^ and the closely related strains using the primer BOX-A1R (5′-CTACGGCAAGGCGACGCTGACG-3′) (Versalovic et al., 1991; Lee et al., 2014c). The BOX-PCR cycling parameters were 5 min at 94°C for pre-denaturation, 35 cycles each of 30 s at 94°C for denaturation, 30 s at 53°C for annealing, 7 min at 65°C for extension and a final extension at 65°C for 8 min (Lee et al., 2014d). The PCR products were visualized by 2% agarose gel electrophoresis.

The protocol of Cashion et al. (1977) was used for the extraction of genomic DNA for DNA-DNA hybridization of strain MUSC 149^T^, *S. graminisoli* NBRC 108883^T^, *S. gramineus* NBRC 107863^T^ and *S. rhizophilus* NBRC 108885^T^. DNA–DNA hybridization was carried out by the Identification Service of the DSMZ, Braunschweig, Germany following the protocol of De Ley et al. (1970) under consideration of the modifications described by Huss et al. (1983). The G + C content of strain MUSC 149^T^ was determined by HPLC (Mesbah et al., 1989).

### Phenotypic Characteristics

**FIGURE 2:**
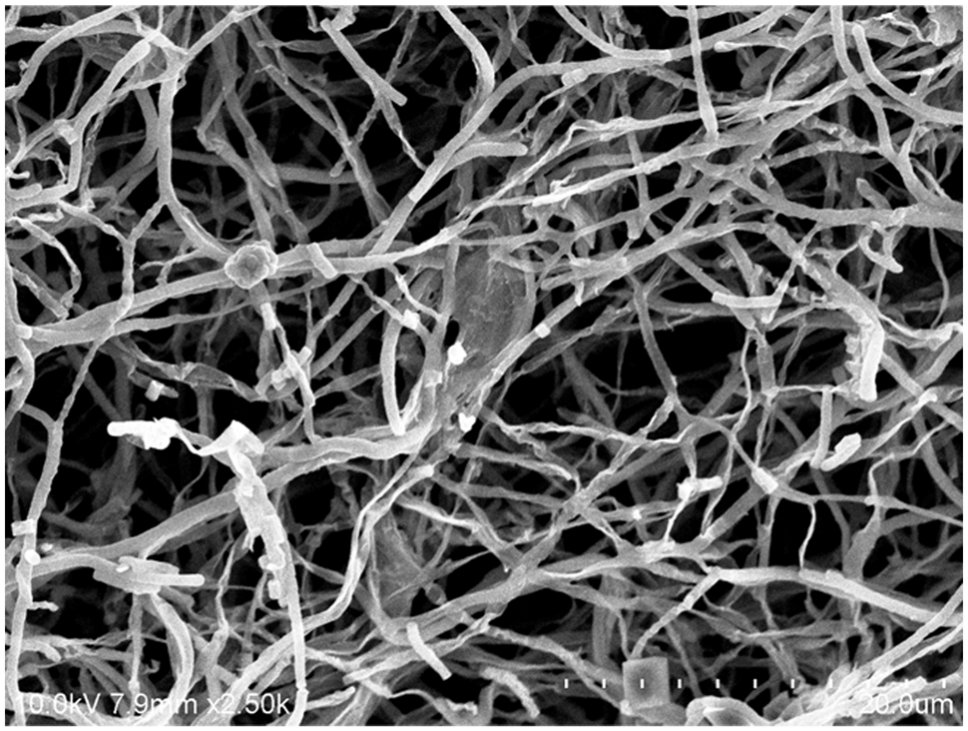
**Scanning electron microscope of *Streptomyces mangrovisoli* MUSC 149^T^**.

The cultural characteristics of strain MUSC 149^T^ were determined following growth on ISP 2, ISP 3, ISP 4, ISP 5, ISP 6, ISP 7 (Shirling and Gottlieb, 1966), actinomycetes isolation agar (AIA; Atlas, 1993), *Streptomyces* agar (SA; Atlas, 1993), starch casein agar (SCA; Küster and Williams, 1964), and nutrient agar (Macfaddin, 2000) for 14 days at 28°C. Light microscopy (80i, Nikon) and scanning electron microscopy (JEOL-JSM 6400) were used to observe the morphology of the strain after incubation on ISP 2 agar at 28°C for 7–14 days (**Figure 2**). The designation of colony color was determined by using the *ISCC-NBS* color charts (Kelly, 1964). Gram staining was performed by standard Gram reaction and confirmed by using KOH lysis (Cerny, 1978). The growth temperature range was tested at 4-40 °C at intervals of 4 °C on ISP 2 agar. The pH range for growth was tested in tryptic soy broth (TSB) between pH 2.0 and 10.0 at intervals of 1 pH unit. The NaCl tolerance was tested in TSB and salt concentrations ranging from 0 to 10% (w/v) at intervals of 2%. The responses to temperature, pH and NaCl were observed for 14 days. Catalase activity and production of melanoid pigments were determined following protocols described by Lee et al. (2014e). The production of melanoid pigments was examined using ISP 7 medium. Hemolytic activity was assessed on blood agar medium containing 5% (w/v) peptone, 3% (w/v) yeast extract, 5% (w/v) NaCl, and 5% (v/v) horse blood (Carrillo et al., 1996). The plates were examined for hemolysis after incubation at 28°C for 7–14 days. Amylolytic, cellulase, chitinase, lipase, protease, and xylanase activities were determined by growing cells on ISP 2 agar and following protocols as described by Meena et al. (2013). The presence of clear zones around the colonies was taken to indicate the potential of isolates for surfactant production. Antibiotic susceptibility tests were performed by the disk diffusion method as described by Shieh et al. (2003). Antimicrobials used and their concentrations per disk (Oxoid, Basingstoke, UK) were as follows: ampicillin (10 μg), ampicillin sulbactam (30 μg), cefotaxime (30 μg), cefuroxime (30 μg), cephalosporin (30 μg), chloramphenicol (30 μg), ciprofloxacin (10 μg), erythromycin (15 μg), gentamicin (20 μg), nalidixic acid (30 μg), Penicillin G (10 μg), streptomycin (10 μg), tetracycline (30 μg), and vancomycin (30 μg). Carbon-source utilization and chemical sensitivity assays were determined using Biolog GenIII MicroPlates (Biolog, USA) according to the manufacturer’s instructions. All of the phenotypic assays mentioned were performed concurrently for strain MUSC 149^T^, *S. graminisoli* NBRC 108883^T^, *S. gramineus* NBRC 107863^T^, and *S. rhizophilus* NBRC 108885^T^.

### Chemotaxonomic Characteristics

The analyses of peptidoglycan amino acid composition and sugars of strain MUSC 149^T^ were carried out by the Identification Service of the DSMZ using protocols of Schumann (2011). Major diagnostic cell wall sugars of strain MUSC 149^T^ were obtained as described by Whiton et al. (1985) and analyzed by TLC on cellulose plates (Staneck and Roberts, 1974). Analysis of respiratory quinones, polar lipids (Kates, 1986) and fatty acids (Sasser, 1990) were carried out by the Identification Service of the DSMZ.

### Extract preparation of MUSC 149^T^

MUSC 149^T^ was grown in TSB for 14 days prior to fermentation process. The fermentation medium used was FM3 (Hong et al., 2009; Lee et al., 2012a). The medium was autoclaved at 121°C for 15 min prior to experiment. Fermentation was carried out in test tubes (30 mm × 200mm) containing 20 mL of FM3, at an angle of 45° for 7–10 days at 28°C. The resulting FM3 medium was recovered by centrifugation at 12000 *g* for 15 min. The supernatant was filtered and subjected to freeze dry process. Upon freeze-drying, the sample was extracted with methanol for 72 h and the methanol-containing extract was filtered and collected. The residue was re-extracted under the same condition twice at 24 h interval. Subsequently, the methanol-containing extract was evaporated using rotary vacuum evaporator at 40°C. The extract of MUSC 149^T^ was collected and suspended in dimethyl sulphoxide (DMSO) as vehicle reagent prior to assay.

### Determination of Antioxidant Activity of MUSC 149^T^ Extract using 2,2-diphenyl-1- picrylhydrazyl (DPPH) Radical Scavenging Method

The stable radical 2,2-diphenyl-1-picrylhydrazyl (DPPH; Sigma–Aldrich) was used to examine antioxidant activity by measuring its hydrogen donating or radical scavenging ability. Scavenging activity on DPPH free radicals by MUSC 149^T^ extract was accessed following previous method with minor modification (Ling et al., 2009). The decrease in radical is measured as decrease in the absorbance of 515 nm. Volume of 195 μL of 0.016% DPPH ethanolic solution was added to 5 μL of extract solution to make up final volume of 200 μL. Gallic acid was included as positive control. Reactions were carried out at room temperature in dark for 20 min before measurement with spectrophotometer at 515 nm. DPPH scavenging activity was calculated as follows:

$$DPPH\,\;\;\;scavenging\,\;\;activity\,\;=\;\frac{Absorbance\,\;\;of\,control-\;Absorbance\,\;\;of\,\;\,sample}{\;Absorbance\,\;\;of\,control}\,\;\times \;100\%$$

### Gas Chromatography–Mass Spectrometry (GC–MS) Analysis

Gas chromatography–mass spectrometry (GC–MS) analysis was performed in accordance with our previous developed method with slight modification (Supriady et al., 2015). The machine used was Agilent Technologies 6980N (GC) equipped with 5979 Mass Selective Detector (MS), HP-5MS (5% phenyl methyl siloxane) capillary column of dimensions 30.0 m × 250 μm × 0.25 μm and used helium as carrier gas at 1 mL/min. The column temperature was programmed initially at 40°C for 10 min, followed by an increase of 3°C/min to 250°C and was kept isothermally for 5 min. The MS was operating at 70 eV. The constituents were identified by comparison of their mass spectral data with those from NIST 05 Spectral Library.

## Results and Discussion

### Phenotypic, Phylogenetic, and Genomic Analyses

Strain MUSC 149^T^ was observed to grow well on ISP 2, ISP 3, ISP 5, ISP 6, ISP 7 agar, actinomycetes isolation agar, starch casein agar, and nutrient agar after 7–14 days at 28°C, and to grow poorly on *Streptomyces* agar, and did not grow on ISP 4 medium. The colors of the aerial and substrate mycelium were media-dependent (Supplementary Table S1). The morphological observation of a 15-day-old culture grown on ISP 2 agar revealed a smooth spore surface and abundant growth of both aerial and vegetative hyphae, which were well developed and not fragmented. These morphological features are consistent with grouping of the strain to the genus *Streptomyces* (Williams et al., 1989). Growth occurred at pH 5.0–8.0 (optimum pH 6.0–7.0), with 0–4% NaCl tolerance (optimum 0–2%) and at 24–36°C (optimum 28–32°C). Cells were found to be positive for catalase but negative for both melanoid pigment production and hemolytic activity. Hydrolysis of carboxymethylcellulose was found to be positive, but negative for hydrolysis of casein, chitin, soluble starch, tributyrin (lipase), and xylan. Strain MUSC 149^T^ can be differentiated from closely related members of the genus *Streptomyces* using a range of phenotypic properties (**Table 1**). In chemical sensitivity assays, cells are resistant to aztreonam, D-serine, fusidic acid, guanine HCl, lincomycin, lithium chloride, minocycline, nalidixic acid, niaproof 4, potassium tellurite, rifamycin RV, sodium bromate, sodium butyrate, 1% sodium lactate, tetrazolium blue, tetrazolium violet, troleandomycin, and vancomycin.

**Table 1:** Differentiation characteristics of strain MUSC 149^T^ and type strains of phylogenetically closely related species of the genus *Streptomyces*.

Characteristics	1	2	3	4
**Morphology (on ISP 2)**				
Color of aerial mycelium	Pale yellow	Grayish yellow	Yellowish white	Light greenish yellow
Color of substrate mycelium	Grayish yellow	Grayish yellow	Pale orange yellow	Grayish yellow
**Growth at**				
24°C	(+)	+	+	(+)
36°C	(+)	(+)	(+)	+
pH 5	(+)	(+)	(+)^γ^	(+)
pH 8	(+)	+	(+)^γ^	(+)
4% NaCl	(+)	+	(+)^γ^	(+)
Catalase	+	+	+	-
Hemolytic	-	-	-	-
**Hydrolysis of**				
Casein (protease)	-	-	+	-
Tributyrin (lipase)	-	-	+	+
Starch (amylolytic)	-	+	+^γ^	+
Carboxymethylcellulose (cellulase)	+	+	-	+
Xylan (xylanase)	-	-	-	+
**Carbon source utilization**				
D-trehalose	+	-	+	-
D-cellobiose	+	-	+	+
α-D-lactose	+	-	+	-
β-methyl-D-glucoside	+	-	-	+
*N*-acetyl-β-D-mannosamine	+	-	-	-
*N*-acetyl-D-galactosamine	+	-	-	-
*N*-acetyl-neuraminic acid	+	-	-	-
D-mannose	-	+	+	+
3-methyl glucose	-	+	-	-
Inosine	+	-	+	-
D-mannitol	+	-	+	+
D-serine	-	+	-	-
Glycyl-L-proline	-	+	+	+
L-alanine	+	-	+	+
L-arginine	-	+	+	+
L-pyroglutamic acid	-	+	+	+
D-gluconic acid	-	+	+	+
Mucic acid	+	-	+	-
Quinic acid	-	-	+	+
D-saccharic acid	-	+	+	-
D-lactic acid methyl ester	-	+	+	+
D-malic acid	+	-	+	+
**Chemical sensitivity assays**				
Troleandomycin	+	+	-	-
Lithium chloride	+	+	-	-

*Strains: 1, S. mangrovisoli sp. nov. MUSC 149^T^; 2, S. rhizophilus NBRC 108885^T^; 3, S. gramineus NBRC 107863^T^; 4, S. graminisoli NBRC 108883^T^. All data were obtained concurrently in this study. +, Positive; –, negative; (+), weak. All strains are positive for utilization of Dextrin, D-maltose, gentiobiose, D-melibiose, α-D-glucose, D-fructose, D-galactose, L-fucose, L-rhamnose, gelatine, L-serine, pectin, oxp-hydroxy-phenylacetic acid, methyl pyruvate, L-malic acid, bromo-succinic acid, tween 40, oxγ-amino-butyric acid, α- hydroxy-butyric acid, oxβ-hydroxy-D,L-butyric acid and oxα-keto-butyric acid. S. graminisoli NBRC 108883^T^. ^γ^Results in accordance with that published for S. gramineus NBRC 107863^T^ by Lee et al. (2012b)*.

The nearly complete 16S rRNA gene sequence was obtained for strain MUSC 149^T^ (1487 bp; GenBank/EMBL/DDBJ accession number KJ632664) and phylogenetic trees were reconstructed to determine the phylogenetic position of this strain (**Figure 1**; Supplementary Figure S1). Phylogenetic analysis exhibited that strain MUSC 149^T^ is closely related to *S. rhizophilus* JR-41^T^, as they formed a distinct clade (**Figure 1**; Supplementary Figure S1). The type strain *S. rhizophilus* JR-41^T^ was isolated from a bamboo (*Sasa borealis*) rhizosphere soil (Lee and Whang, 2014). The 16S rRNA gene sequence analysis of strain MUSC 149^T^ showed the highest similarity to that of *S. rhizophilus* NBRC 108885^T^ (99.2% sequence similarity), followed by *S. gramineus* NBRC 107863^T^ (98.7%) and *S. graminisoli* NBRC 108883^T^ (98.5%); sequences similarities of less than 98.3% were obtained with the type strains of other species of the genus *Streptomyces*. The DNA–DNA hybridization values between strain MUSC 149^T^ and *S. rhizophilus* NBRC 108885^T^ (12.4 ± 3.3%), followed by *S. gramineus* NBRC 107863^T^ (13.7 ± 0.5%) and *S. graminisoli* NBRC 108883^T^ (27.3 ± 1.9%) were significantly below 70%, the threshold value for the delineation of bacterial species (Wayne et al., 1987). The BOX-PCR results indicated that strain MUSC 149^T^ yielded a unique BOX-PCR fingerprint compared with the closely related type strains (Supplementary Figure S2). These results are in agreement with results of DNA–DNA hybridizations, which indicate that strain MUSC 149^T^ represents a novel species.

### Chemotaxonomic Analyses

Chemotaxonomic analyses showed that the cell wall of strain MUSC 149^T^ is of cell-wall type I (Lechevalier and Lechevalier, 1970) as it contains LL-diaminopimelic. The presence of LL-diaminopimelic has been observed in many other species of the genus *Streptomyces* (Lee et al., 2005, 2014b; Xu et al., 2009; Hu et al., 2012; Ser et al., 2015). The predominant menaquinones of strain MUSC 149^T^ were identified as MK-9(H_8_) (59%) and MK-9(H_6_) (15%). This is in agreement with Kim et al. (2003) that the predominant menaquinones of members of the genus *Streptomyces* are MK-9(H_6_) and MK-9(H_8_). The cell wall sugars detected were glucose, mannose and ribose. Strain MUSC149^T^ shared the same sugar profile with *S. gilvigriseus* (Ser et al., 2015). Furthermore the sugars glucose and ribose were detected in other members of the genus *Streptomyces* such as *S. rhizophilus* JR-41^T^, *S. graminisoli* JR-19^T^ (Lee and Whang, 2014), *S. gramineus* JR-43^T^ (Lee et al., 2012b), *S. shenzhenensis* 172115^T^ (Hu et al., 2011), and *S. pluripotens* (Lee et al., 2014b). The G + C content of strain MUSC 149^T^ was determined to be 72.7 mol%; this is within the range of 67.0–78.0 mol% described for species of the genus *Streptomyces* (Kim et al., 2003).

**FIGURE 3:**
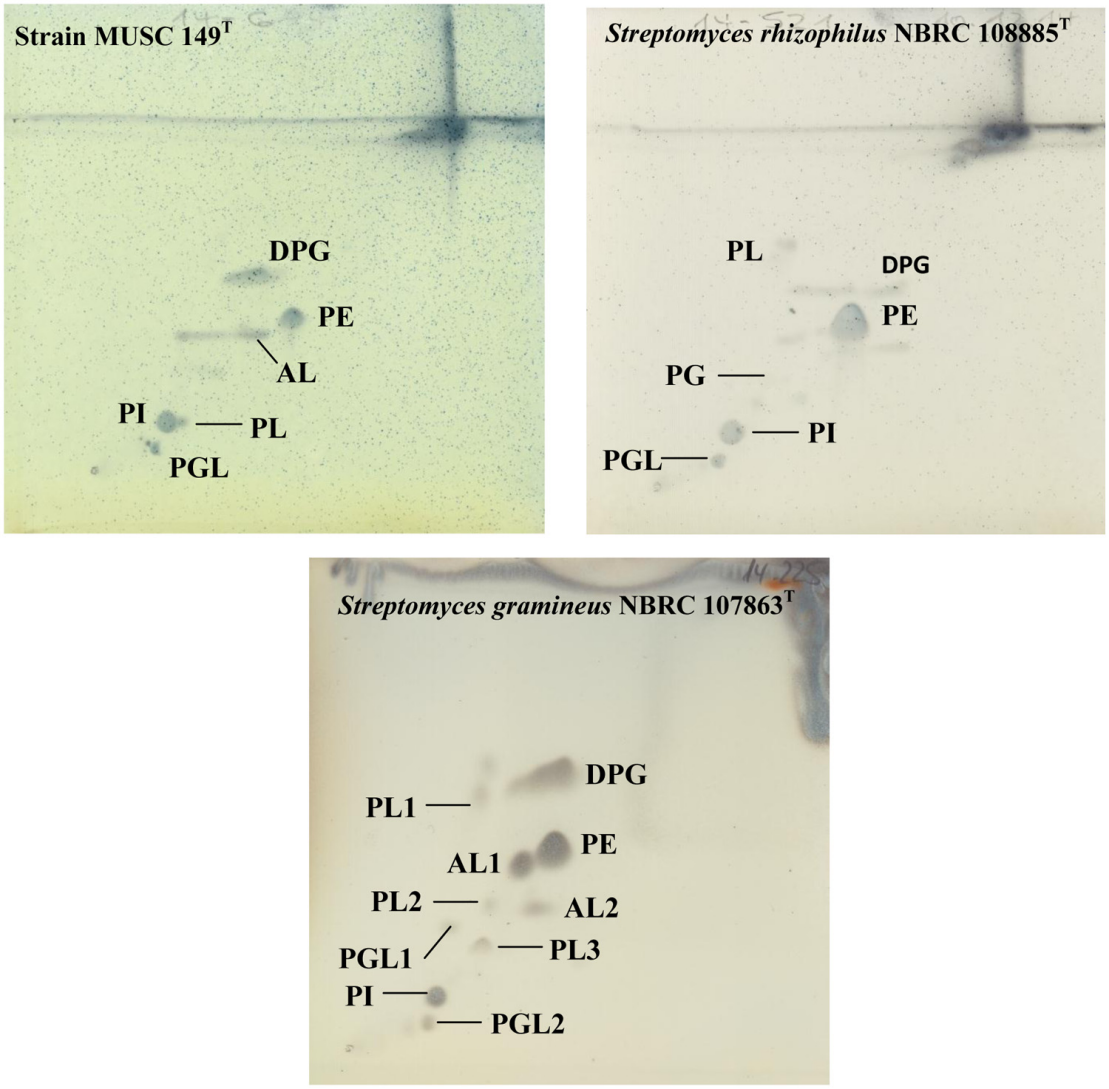
**Two dimensional total lipid profile of strain MUSC 149^T^, *S. rhizophilus* NBRC 108885^T^ and *S. gramineus* NBRC 107863^T^**. AL, Aminolipid; DPG, Diphosphatidylglycerol; PL, Phospholipid; PI, Phosphatidylinositol; PE, Phosphatidylethanolamine; PG, Phosphatidylglycerol; PGL, Phosphoglycolipid.

The polar lipid analysis showed the presence of aminolipid, diphosphatidylglycerol, phosphatidylethanolamine, phosphatidylinositol, phosphoglycolipid, and phospholipid (**Figure 3**). Differences in polar lipid profiles indicated that MUSC 149^T^ is different from related type strains (**Figure 3**); for example, strain MUSC 149^T^ was found to contain aminolipid, lipid that was not detected in *S. rhizophilus* NBRC 108885^T^ (**Figure 3**). The fatty acids profiles of strain MUSC 149^T^ and closely related type strains are given shown in **Table 2**.

**Table 2:** Cellular fatty acid composition of strain MUSC 149^T^ and its closely related *Streptomyces* species.

Fatty acid	1	2	3	4
iso-C_12:0_	0.1	–	–	0.1
C_12:0_	0.1	–	–	0.1
iso-C_13:0_	0.4	0.6	0.2	0.4
anteiso-C_13:0_	0.5	0.3	0.2	0.3
iso-C_14:0_	2.9	3.5	4.3	5.7
C_14:0_	0.6	0.5	0.2	1.1
iso-C_15:0_	17.7	18.3	19.1	12.5
anteiso-C_15:0_	26.2	26.5	21.4	17.5
C_15:0_w6c	–	–	–	0.3
C_15:0_	1.8	1.5	–	2.1
iso-C_16:1_ H	1.9	–	1.3	1.7
iso-C_16:0_	16.0	15.4	19.2	25.1
C_16:1_Cis9	2.8	–	–	–
C_16:0_	4.0	9.4	4.3	7.9
iso-C_17:1_w9c	–	1.3	5.0	3.1
anteiso-C_17:1_w9c	2.4	0.6	1.9	1.9
iso-C_17:0_	6.1	10.3	10.4	5.0
anteiso-C_17:0_	11.3	10.7	9.6	9.2
C_17:1_w8c	–	–	0.4	0.4
C_17:0_ CYCLO	0.4	–	0.5	0.6
C_17:0_	0.3	0.7	0.7	0.5

*Strains: 1, S. mangrovisoli sp. nov. MUSC 149^T^; 2, S. rhizophilus NBRC 108885^T^; 3, S. gramineus NBRC 107863^T^; 4, S. graminisoli NBRC 108883^T^. –, <0.1% or not detected. All data are obtained concurrently from this study*.

The major cellular fatty acids in MUSC 149^T^ were identified as anteiso-C_15:__0_ (26.2%), iso-C_15:0_ (17.7%), iso-C_16:0_ (16.0%) and anteiso-C_17:0_ (11.3%). The fatty acids profile of MUSC 149^T^ is consistent with those of closely related phylogenetic neighbors such as *S. rhizophilus* NBRC 108885^T^, *S. gramineus* NBRC 107863^T^, and *S. graminisoli* NBRC 108883^T^, which contain anteiso-C_15:0_ (26.5–17.5%), iso-C_16:0_ (25.1–15.4%), and iso-C_15:0_ (18.3–12.5%) as their major fatty acids (**Table 2**). However, the fatty acid profile of MUSC 149^T^ was quantitatively different from those of these type strains; for example, although anteiso-C_15:0_ (26.2%) was found to be predominant in strain MUSC 149^T^, the amount of anteiso-C_15:0_ was significantly lesser (17.5%) in *S. graminisoli* NBRC 108883^T^ (**Table 2**).

Based on the results of DNA-DNA hybridization, phylogenetic analysis, chemotaxonomic, phenotypic and DNA fingerprinting, strain MUSC 149^T^ merits assignment to a novel species in the genus *Streptomyces*, for which the name *S. mangrovisoli* sp. nov. is proposed.

### Antioxidant Activity of MUSC 149^T^ Extract

The antioxidant evaluation assay DPPH is based upon the reduction of DPPH free radical. It is widely used to determine free radical scavenging capacity of the tested samples (Blois, 1958; Molyneux, 2004). As a free radical, DPPH is observed as purple solution when dissolved in appropriate solvent. It is known to exhibit a high absorption at 515 nm when measured with visible spectroscopy. In the presence of free radical-scavenging agent(s) or hydrogen donor(s), the odd electron of DPPH will be paired off, it will subsequently result in discoloration of solution to become either yellowish or colorless. The strength of the radical scavenging or anti-oxidant activity can then be quantified by the difference of absorbance obtained with the samples when is comparing to control.

**FIGURE 4:**
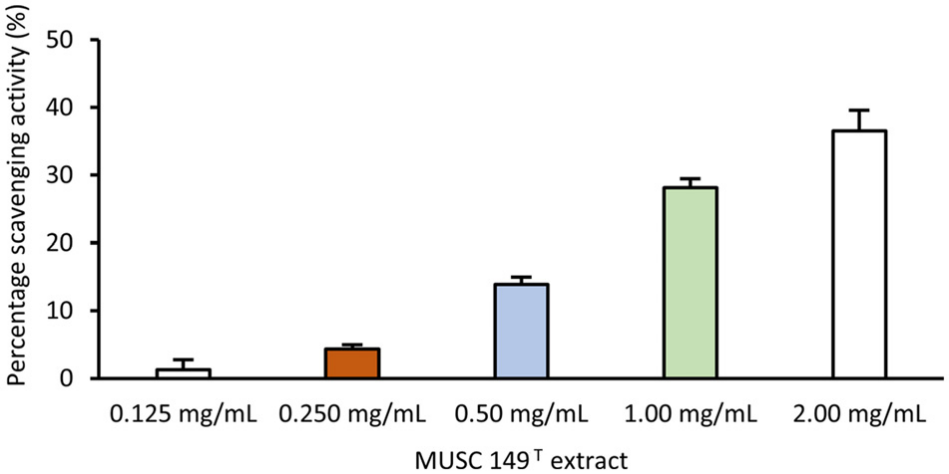
**Antioxidant activity of MUSC 149^T^ methanolic extract.** Antioxidant activity of MUSC 149^T^ was evaluated at different concentration and values are SEM of four replicates.

The DPPH scavenging assay was employed to examine the antioxidant activity of MUSC 149^T^ extract. The extract was tested for a dose-response study with five different concentrations (0.125, 0.25, 0.5, 1.0, and 2.0 mg/mL). Based on the results obtained, the extract of MUSC 149^T^ displayed a dose-dependent manner of antioxidant activity. It was inferred by a gradual increase in scavenging activity of MUSC 149^T^ extract with a low concentration of extract at 0.125 mg/mL to the highest concentration at 2.0 mg/mL. The scavenging activity of lowest concentration at 0.125 mg/mL and the highest concentration at 2.0 mg/mL was recorded at 1.1 ± 1.4% and 36.5 ± 3.0%, respectively (**Figure 4**). The ability of MUSC 149^T^ extract to scavenge DPPH free radicals indicates the possible presence of antioxidant agent(s) in the tested MUSC 149^T^ extract.

### GC–MS Analysis of MUSC 149^T^ Methanolic Extract

Growing evidence implies that the accumulation of free radicals may contribute to pathogenesis of chronic diseases including Parkinson’s disease and various types of cancers (Floyd and Hensley, 2002; Farooqui and Farooqui, 2009; Goldkorn et al., 2014; Mahalingaiah and Singh, 2014). Synthetic antioxidants may be able to scavenge these notorious free radicals, however, currently available antioxidants display low solubility and may promote negative health impacts (Barlow, 1990; Panicker et al., 2014). With this in mind, the search of the antioxidants from natural resources has always been one of the major focuses for many researchers (Lee et al., 1998; Harvey et al., 2015). In order to explore this premise, we examined the antioxidant activity of the extract of MUSC 149^T^. The results obtained demonstrated that MUSC 149^T^ extract was posing significant antioxidant activity. This has prompted the necessities to further examine the chemical constituents which present in the extract of MUSC 149^T^.

**FIGURE 5:**
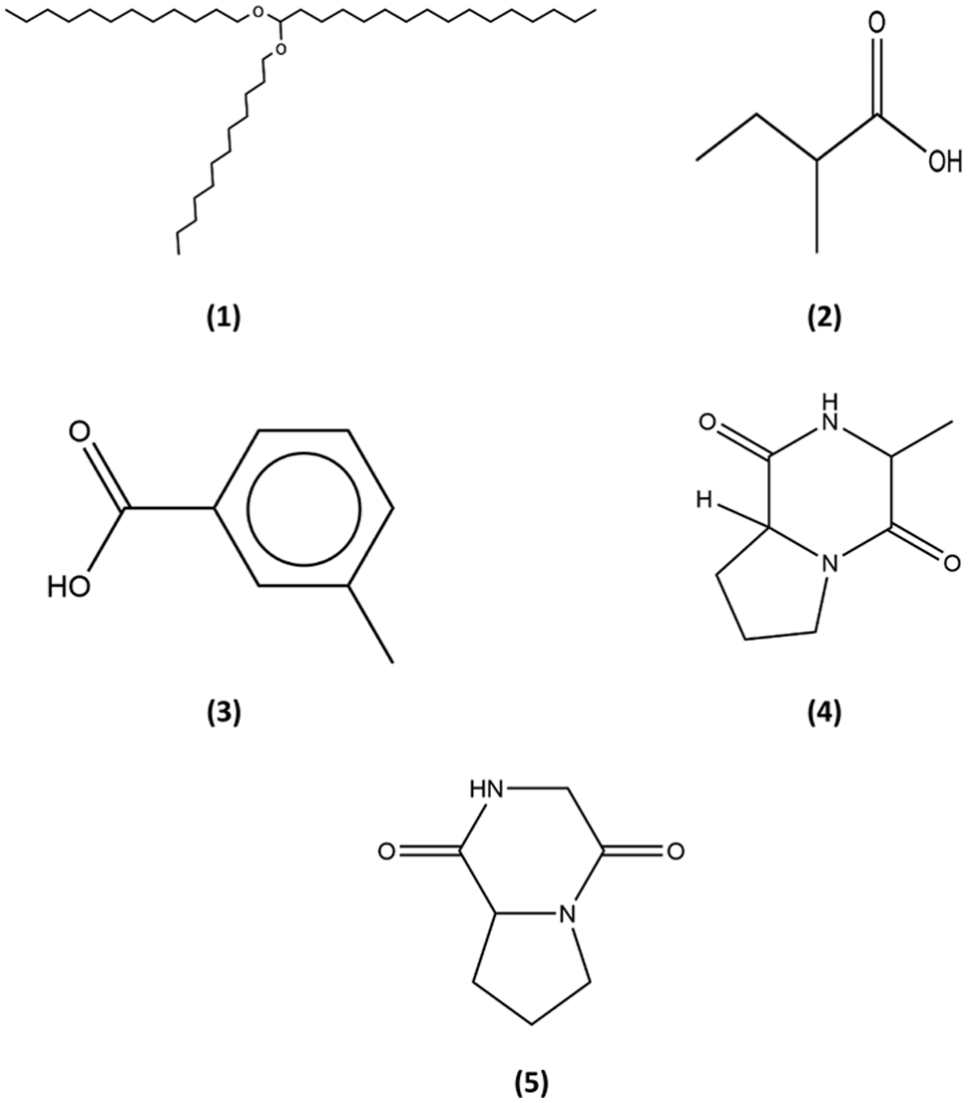
**Chemical structures of the identified compounds from MUSC 149^T^.** (1) Hexadecane, 1,1-bis(dodecyloxy); (2) Butanoic acid, 2-methyl-; (3) Benzoic acid, 3-methyl-; (4) (3R,8aS)-3-methyl-1,2,3,4,6,7,8,8a-octahydropyrrolo[1,2-a]pyrazine-1,4-dione; (5) Pyrrolo[1,2-a]pyrazine-1,4-dione, hexahydro-.

As *Streptomyces* are known to produce various secondary metabolites with diverse biological activity, numerous studies have incorporated powerful analytical techniques such as GC–MS to assist with the chemical analysis (Pollak and Berger, 1996; Karanja et al., 2010; Sudha and Masilamani, 2012; Ara et al., 2014; Jog et al., 2014). This robust technique produces reliable results as it combines separation power of GC and detection power of MS by generating characteristic mass spectral fragmentation patterns for each compounds present in mixture (Hites, 1997). For instance, recent study by Kim et al. (2008) has described detection of the bioactive compound (protocatechualdehyde) present in the extract of *S. lincolnensis* M-20 by using the GC–MS. With this intention, GC–MS analysis was performed in this study to explore the chemical constituents present in the extract of MUSC 149^T^. Using this analytical technique, we have identified chemical constituents of the extract of MUSC 149^T^ (**Table 3**) and the chemical structures (**Figure 5**) as Hexadecane, 1,1-bis(dodecyloxy) (1), Butanoic acid, 2-methyl- (2), Benzoic acid, 3-methyl- (3) (3R,8aS)-3-methyl-1,2,3,4,6,7,8,8a-octahydropyrrolo[1,2-a]pyrazine-1,4-dione (4), and Pyrrolo[1,2-a]pyrazine-1,4-dione, hexahydro- (5).

**Table 3:** Compounds identified from MUSC 149^T^ extract through Gas chromatography–mass spectrometry (GC–MS).

No.	Retention time	Compound	Formula	Molecular weight	Similarity (%)
1	5.913	Hexadecane, 1,1-bis(dodecyloxy)	C_40_H_82_O_2_	595	64
2	9.753	Butanoic acid, 2-methyl-	C_5_H_10_O_2_	102	74
3	33.499	Benzoic acid, 3-methyl-	C_8_H_8_O_2_	136	90
4	51.535	(3R,8aS)-3-methyl-1,2,3,4,6,7,8,8a-octahydropyrrolo [1,2-a]pyrazine-1,4-dione	C_8_H_12_N_2_O_2_	168	90
5	52.994	Pyrrolo [1,2-a]pyrazine-1,4-dione, hexahydro-	C_7_H_10_N_2_O_2_	154	90

The detection of heterocyclic organic compound in extract is deemed as one of the most important findings in current study. Pyrrolizidines are widely present or synthesized in several marine *Streptomyces* species (Olano et al., 2009b; Robertson and Stevens, 2014). Furthermore, pyrrolizidines are known to exhibit a wide range of bioactivities which including antitumor, anti-angiogenesis, and antioxidant activities. For instance, the detection of the compound known as pyrrolo[1,2-a]pyrazine-1,4-dione, hexahydro- (**Table 3**; **Figure 5**) in the extract has suggested the antioxidant activity could be contributed by this compound. Furthermore, other recent findings conducted on this compound suggested strong antioxidant activities as well (Gopi et al., 2014; Balakrishnan et al., 2015). These findings have demonstrated that pyrrolo[1,2-a]pyrazine-1,4-dione, hexahydro- was able to scavenge or reduce amount of free radicals as evaluated by using reducing power assay. In short, an antioxidant is likely to play important roles in prevention and treatment of chronic diseases (Morales-González, 2013). The strong free radical scavenging effect possessed by the extract of MUSC 149^T^ warrants the future investigations into different type of biological activities.

### Description of *S. mangrovisoli* sp. nov.

*Streptomyces mangrovisoli* sp. nov. (man.gro.vi.so′li. N.L. n. mangrovum, mangrove; L. gen. n. soli, of soil; N.L. gen. n. mangrovisoli, of mangrove soil, referring to the source of the inoculum).

Cells stain Gram-positive and form pale yellow aerial and grayish yellow substrate mycelium on ISP 2 agar. The colors of the aerial and substrate mycelium are media-dependent (Supplementary Table S1). Grows well on ISP 2, ISP 3, ISP 5, ISP 6, ISP 7 agar, actinomycetes isolation agar, starch casein agar, and nutrient agar after 1–2 weeks at 28°C; and to grow poorly on *Streptomyces* agar, whereas no growth on ISP 4 medium. Grows occur at pH 5.0–8.0 (optimum pH 6.0–7.0), with 0–4% NaCl tolerance (optimum 0–2%) and at 24–36°C (optimum 28–32°C). Cells are positive for catalase but negative for both melanoid pigment production and hemolytic activity. Carboxymethylcellulose is hydrolysed but negative for hydrolysis of casein, chitin, soluble starch, tributyrin (lipase), and xylan. The following compounds are utilized as sole carbon sources: acetic acid, acetoacetic acid, α-D-glucose, α-D-lactose, α-hydroxy-butyric acid, α-keto-butyric acid, α-keto-glutaric acid, β-hydroxyl-D,L-butyric acid, β-methyl-D-glucoside, bromo-succinic acid, citric acid, D-cellobiose, Dextrin, D-fructose, D-fructose-6-phosphate, D-fucose, D-galactose, D-galacturonic acid, D-gluconic acid, D-glucose-6-phosphate, D-glucuronic acid, D-lactic acid methyl ester, D-malic acid, D-maltose, D-mannitol, D-melibiose, D-raffinose, D-saccharic acid, D-sorbitol, D-trehalose, D-turanose, formic acid, gelatin, gentiobiose, glucuronamide, inosine, L-fucose, L-galactonic acid lactone, L-lactic acid, L-malic acid, L-rhamnose, methyl pyruvate, mucic acid, *N*-acetyl-β-D-mannosamine, *N*-acetyl-D-galactosamine, *N*-acetyl-D-glucosamine, *N*-acetyl-neuraminic acid, pectin, *p*-hydroxyl-phenylacetic acid, propionic acid, quinic acid, stachyose, sucrose, Tween 40, and γ-amino-butyric acid. The following compounds are not utilized as sole carbon sources: D-salicin, D-mannose, D-arabitol, myo-inositol, glycerol, D-aspartic acid, D-serine, glycyl-L-proline, and 3-methyl glucose. L-alanine, L-histidine, and L-serine are utilized as sole nitrogen sources. L-arginine, L-aspartic acid, L-glutamic acid, and L-pyroglutamic acid are not utilized as sole nitrogen sources. Extract of the type strain exhibits strong antioxidant activity in a dose-dependent manner. The G + C content of the genomic DNA of the type strain is 72.7 mol%.

The type strain is MUSC 149^T^ (=MCCC 1K00699^T^=DSM 100438^T^), isolated from mangrove soil collected from the Tanjung Lumpur mangrove forest located in the state of Pahang, Peninsular Malaysia. The 16S rRNA gene sequence of strain MUSC 149^T^ has been deposited in GenBank/EMBL/DDBJ under the accession number KJ632664.

## Conflict of Interest Statement

The authors declare that the research was conducted in the absence of any commercial or financial relationships that could be construed as a potential conflict of interest.
