# Supplementary material for: Presence of antioxidative agent, Pyrrolo[1,2-a]pyrazine-1,4-dione, hexahydro- in newly isolated Streptomyces mangrovisoli sp. nov
Source: Front Microbiol. 2015 Aug 20;6:854. doi: 10.3389/fmicb.2015.00854 (PMC4542459; doi:10.3389/fmicb.2015.00854)
Supplement: Supplementary file 1 [file Data_Sheet_1.DOC]

**Fig. S1.** Neighbour-joining tree (Saitou and Nei 1987) based on 1487 nucleotides of 16S rRNA gene sequence showing the relationship between strain MUSC 149^T^ and representatives of related taxa. Bootstrap values (>50%) based on 1000 re-sampled datasets are shown at branch nodes. Bar, 0.002 substitutions per site. Asterisks indicate that the corresponding nodes were also recovered using maximum-likelihood tree-making algorithms.

*Streptomyces bungoensis* NBRC 15711^T^ (AB184696)

*Streptomyces galbus* DSM 40089^T^ (X79852)

*Streptomyces longwoodensis* LMG 20096^T^ (AJ781356)

*Streptomyces capoamus* JCM 4734^T^ (AB045877)

*Streptomyces canarius* NBRC 13431^T^ (AB184396)

*Streptomyces curacoi* NRRL B-2901 (EF626595)

*Streptomyces cyaneus* NRRL B-2296^T^ (AF346475)

*Streptomyces kunmingensis* NBRC 14463^T^ (AB184597)

*Streptomyces chartreusis* NBRC 12753^T^ (AB184839)

*Streptomyces lanatus* NBRC 12787^T^ (AB184845)

*Streptomyces psammoticus* NBRC 13971^T^ (AB184554)

*Streptomyces echinatus* NBRC 12763^T^ (AB184126)

*Streptomyces costaricanus* NBRC 100773^T^ (AB249939)

*Streptomyces griseofuscus* NBRC 12870^T^ (AB184206)

*Streptomyces murinus* NBRC 12799^T^ (AB184155)

*Streptomyces graminearus* NBRC 15420^T^ (AB184667)

*Streptomyces graminisoli* JR-19^T^ (HQ267975)

*Streptomyces shenzhenensis* 172115^T^ (HQ660226)

*Streptomyces rhizophilus* JR-41^T^ (HQ267989)

*Streptomyces gramineus* JR-43^T^ (HM748598)

***Streptomyces mangrovisoli* MUSC149^T^ (KJ632664)**

*Streptomyces violarus* NBRC 13104^T^ (AB184316)

*Streptomyces fumigatiscleroticus* NBRC 12999^T^ (AB184248)

*Streptomyces asterosporus* NRRL B-24328^T^ (AY999902)

*Streptomyces calvus* ISP 5010^T^ (AY999780)

*Streptomyces flavoviridis* NBRC 12772^T^ (AB184842)

*Streptomyces pilosus* NBRC 12807^T^ (AB184161)

**100***

**100***

**100***

**82***

*

**98***

**96***

**50***

**65**

**64***

*

*

*

**65**

**55**

*

**57***

**79***

*

**72***

**0.002**

**Hooi-Leng Ser, Uma Devi Palanisamy, Wai-Fong Yin, Sri Nurestri Abd Malek, Kok-Gan Chan, Bey-Hing Goh*, Learn-Han Lee^*^ (2015).** *Streptomyces mangrovisoli* sp. nov., an antioxidant agent-producing streptomycete *Frontiers in Microbiology*

*Correspondence: Lee Learn-Han, E-mail: [lee.learn.han@monash.edu](mailto:lee.learn.han@monash.edu) ; [leelearnhan@yahoo.com](mailto:leelearnhan@yahoo.com). Goh Bey-Hing, E-mail: goh.bey.hing@monash.edu. Affiliation: Monash University Malaysia


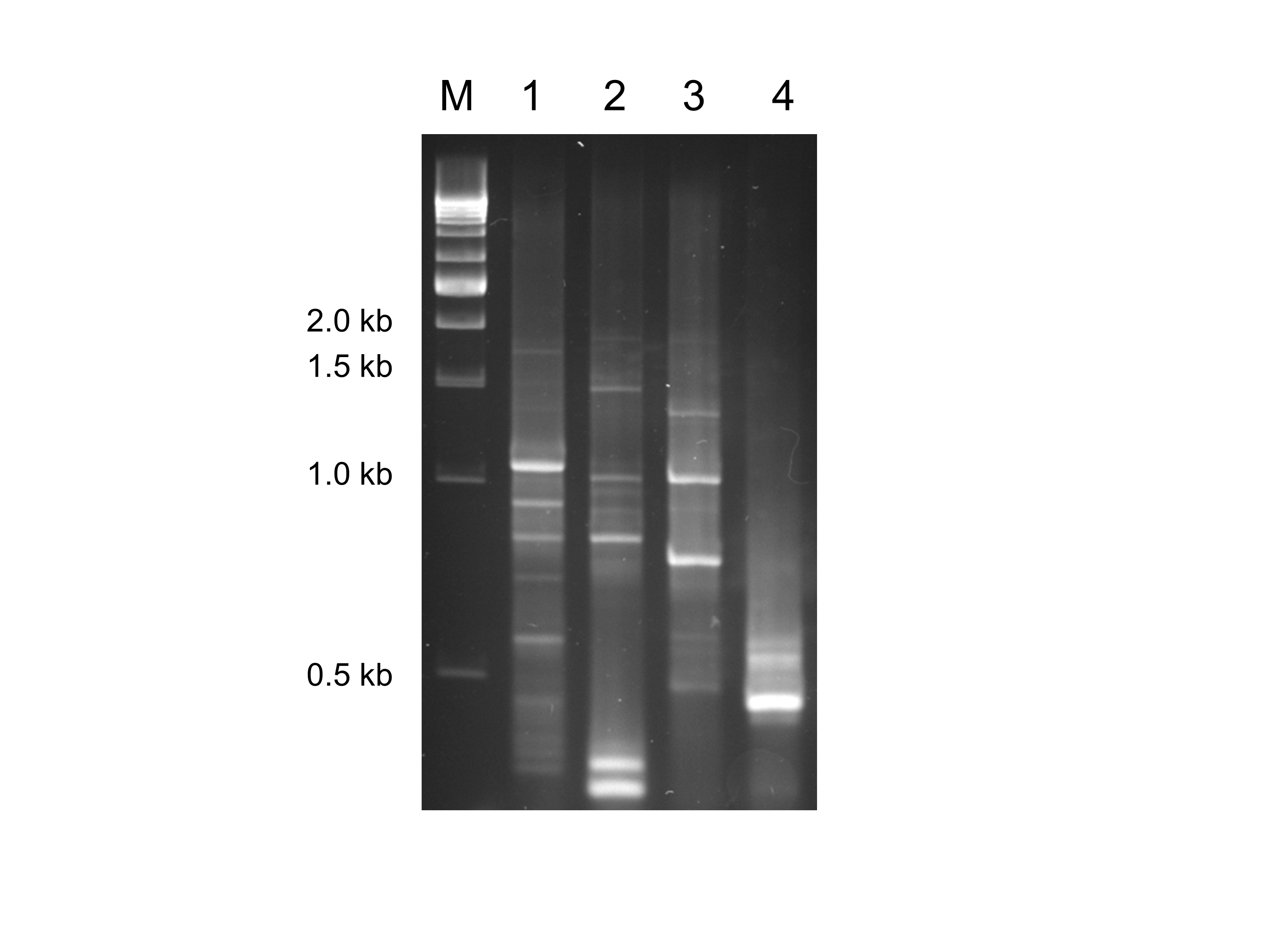
**Fig. S2.** BOX-PCR comparison of strain MUSC 149^T^ and the closest related type strains. Lanes: 1, *Streptomyces mangrovosoli* sp. nov. MUSC 149^T^; 2, *Streptomyces rhizophilus* NBRC 108885^T^; 3, *Streptomyces graminisoli* NBRC 108883^T^; 4, *Streptomyces gramineus* NBRC 107863^T^; M, GeneRuler 1kb DNA ladder marker.

**Hooi-Leng Ser, Uma Devi Palanisamy, Wai-Fong Yin, Sri Nurestri Abd Malek, Kok-Gan Chan, Bey-Hing Goh*, Learn-Han Lee^*^ (2015).** *Streptomyces mangrovisoli* sp. nov., an antioxidant agent-producing streptomycete *Frontiers in Microbiology*

*Correspondence: Lee Learn-Han, E-mail: [lee.learn.han@monash.edu](mailto:lee.learn.han@monash.edu) ; [leelearnhan@yahoo.com](mailto:leelearnhan@yahoo.com). Goh Bey-Hing, E-mail: goh.bey.hing@monash.edu. Affiliation: Monash University Malaysia

**Table S1.** Cultural characteristics of strain MUSC 149^T^ on different media at 28 ^0^C after 7-14 days of incubation.

-, Not detected

| **Medium** | **Growth** | **Colony color** | |
| --- | --- | --- | --- |
|  |  | **Aerial mycelium** | **Substrate mycelium** |
| Yeast malt agar (ISP 2) | Good | Pale yellow | Grayish yellow |
| Oat Meal agar (ISP 3)  Inorganic Salt Starch agar (ISP 4) | Good  No growth | Yellowish White  - | Pale Yellow  - |
| Glycerol Asparagine Agar Base (ISP 5) | Good | Yellowish White | Pale Greenish Yellow |
| Peptone Yeast Extract Iron agar (ISP 6) | Good | Yellowish White | Black |
| Tyrosine agar base (ISP 7) | Good | Yellowish White | Pale Greenish Yellow |
| *Streptomyces* agar | Poor | Pale orange yellow | Pale Yellow |
| Starch casein agar | Good | Pale Yellow | Yellowish White |
| Actinomycete isolation agar | Good | Pale Yellowish Green | Yellowish White |
| Nutrient agar | Good | Light Grayish Yellowish Brown | Brilliant Yellow |

**Hooi-Leng Ser, Uma Devi Palanisamy, Wai-Fong Yin, Sri Nurestri Abd Malek, Kok-Gan Chan, Bey-Hing Goh*, Learn-Han Lee^*^ (2015).** *Streptomyces mangrovisoli* sp. nov., an antioxidant agent-producing streptomycete *Frontiers in Microbiology*

*Correspondence: Lee Learn-Han, E-mail: [lee.learn.han@monash.edu](mailto:lee.learn.han@monash.edu) ; [leelearnhan@yahoo.com](mailto:leelearnhan@yahoo.com). Goh Bey-Hing, E-mail: goh.bey.hing@monash.edu. Affiliation: Monash University Malaysia
